# LncRNA *MALAT*1 Regulates miR-144-3p to Facilitate Epithelial-Mesenchymal Transition of Lens Epithelial Cells via the ROS/NRF2/Notch1/Snail Pathway

**DOI:** 10.1155/2020/8184314

**Published:** 2020-11-12

**Authors:** Wei Ye, Jiyuan Ma, Fang Wang, Tong Wu, Mengmei He, Ji Li, Rui Pei, Luning Zhang, Yafen Wang, Jian Zhou

**Affiliations:** Department of Ophthalmology, Xijing Hospital, Eye Institute of Chinese PLA, Fourth Military Medical University, Xi'an, Shaanxi, China

## Abstract

Diabetic cataract is a common complication of diabetes. The epithelial-mesenchymal transition (EMT) of lens epithelial cells (LECs) is a key event in the development of diabetic cataracts. Metastasis-associated lung adenocarcinoma transcript 1 (*MALAT*1) has been reported to be highly expressed in different tissues of diabetic patients. This study is aimed at investigating the function and mechanism of *MALAT*1 in the regulation of EMT in human LECs under high glucose conditions. *MALAT*1, *α*-smooth muscle actin (*α*-SMA), fibronectin (FN), and nuclear factor erythroid-derived 2-like 2 (NRF2) were highly expressed in the LECs of diabetic cataract patients and in the human LECs under high glucose conditions; meanwhile, the decreased expressions of E-cadherin and zonula occludens 1 (ZO-1) were detected. Knockdown of *MALAT*1 could significantly reduce ROS, prevent EMT, arrest S phase cell cycle, and suppress the expression of total NRF2 and its nucleus translocation in LECs. Furthermore, after *NRF*2 was knocked down, total NRF2, *α*-SMA, and FN in cells, and NRF2, Notch intracellular domain (NICD), and Snail were decreased in the nucleus. Using bioinformatics methods, we predicted that *MALAT*1 and *NRF*2 shared the same microRNA-144-3p (miR-144-3p) combining site. Luciferase reporter coupled with qRT-PCR assays revealed that miR-144-3p was a target of *MALAT*1, which was confirmed to downregulate miR-144-3p in the LECs. In addition, after transfection of miR-144-3p mimics or inhibitor, western blot assay demonstrated that miR-144-3p negatively regulated the expression of total NRF2, *α*-SMA, and FN in cells, and NRF2, NICD, and Snail in the nucleus without affecting Kelch-like ECH-associated protein 1 (KEAP1). Finally, we confirmed that transfection of sh*MALAT*1 inhibited NRF2 expression, and its mediated EMT could be rescued by miR-144-3p inhibitor; transfection of pcDNA3.1-*MALAT*1 promoted NRF2 expression, and its mediated EMT could be reversed by miR-144-3p inhibitor. In summary, we demonstrate that *MALAT*1 regulates miR-144-3p to facilitate EMT of LECs via the ROS/NRF2/Notch1/Snail pathway.

## 1. Introduction

Diabetes is a metabolic disease characterized by elevated blood glucose. Complications of diabetes occur in all organ systems, such as diabetic nephropathy, diabetic neuropathy, diabetic retinopathy, and diabetic cataracts [1–5]. Diabetic patients suffer from cataracts earlier than nondiabetic patients [6], and cataract surgery on diabetic patients may lead to more complications, especially in hyperglycemia conditions [7]. Therefore, it is helpful to explore the pathogenesis of diabetic cataracts in order to find the most effective way to prevent them.

The proportions of cortical and subcapsular cataracts are higher in both type 1 and type 2 diabetic patients with cataracts than the proportions seen in age-related cataracts [8, 9]. Posterior subcapsular cataracts occur because of abnormal cells and extracellular matrix under the lens posterior capsules in humans [10], and diabetic subcapsular cataracts in rats and mice are highly associated with the epithelial-mesenchymal transition (EMT) of lens epithelial cells (LECs) [11, 12]. However, the mechanism of EMT in LECs in diabetic conditions is still unclear.

Long noncoding RNAs (lncRNAs) are defined as RNA molecules of more than 200 nucleotides without protein-coding capacity [13], which function in regulating the processes of apoptosis, autophagy, cell cycle, and EMT [14–16]. *MALAT*1 is a 6.5-knt lncRNA, which was first found in lung adenocarcinoma, playing significant roles in the pathophysiological process of diabetes and diabetes-related complications by modulating gene transcription [17]. It can promote oxidative stress in human LECs under high glucose conditions [16], and reactive oxygen species (ROS) have been reported to induce the EMT process in LECs of diabetic cataracts [18]. The role of *MALAT*1 in modulating ROS and EMT of LECs in diabetic cataracts remains unclear.

KEAP1/NRF2 system is one of the main cellular defense mechanisms against oxidative stress in LECs. When ROS increases, KEAP1 is oxidized and covalently modified, and NRF2 translocates to the nucleus and induces the generation of antioxidant enzymes that protect cells from oxidative stress and apoptosis by reducing ROS accumulation in diabetic cataracts [19–21]. Meanwhile, nucleus translocation of NRF2 can regulate more than 100 genes, including *Notch*1, to promote cell proliferation, differentiation, and migration [22–24]. In hepatocellular carcinoma cells, ROS-induced nucleus translocation of NRF2 can activate the Notch1/Snail signaling pathway to accelerate EMT and metastasis of tumor cells [25]. This research implies that ROS may induce NRF2 nucleus translocation, subsequently trigger the Notch signaling pathway, and result in the EMT of LECs in the formation of diabetic cataracts.

The theory of competing endogenous RNA (ceRNA) proposes that lncRNA and mRNA transcripts can affect each other by competitively combining with a miRNA response element (MRE) to influence posttranscription [26]. *MALAT*1 can regulate target genes by acting as a “sponge” of some miRNAs [27, 28], miR-144-3p in osteosarcoma cells that are regulated by *MALAT*1 function as competing endogenous RNAs (ceRNAs) [29]. It has been reported that miR-144-3p is widely expressed in the tissues of diabetic patients, and that it may negatively regulate *NRF*2 expression in retinal pigment epithelium (RPE) cells, plasma, retinal endothelial cells, and epicardial adipose tissues [30–33]. Taking all of these findings into consideration, we hypothesize that *MALAT*1 may promote ROS expression, induce nucleus translocation of NRF2, and regulate NRF2 expression via a competing endogenous RNA of miR-144-3p.

In the present study, we first confirmed that *MALAT*1 and NRF2 are highly expressed in LECs of diabetic patients with cataracts and in LECs in a hyperglycemic environment. Second, we found that knockdown of *MALAT*1 could reduce the level of ROS, expression of NRF2, nucleus translocation of NRF2, and EMT of LECs in a high-glucose environment. Third, we discovered that knockdown of *NRF*2 reduced EMT by blocking the Notch1/Snail pathway. Most importantly, we found that *MALAT*1 competitively sequestered miR-144-3p and relieved the inhibitory effect of miR-144-3p on NRF2, increasing NRF2 expression. Finally, our study clarified a novel role of *MALAT*1 in regulating miR-144-3p, ROS, and then activating the NRF2/Notch1 pathway to facilitate diabetic cataracts, and suggested that *MALAT*1 might be a target for the prevention of diabetic cataracts.

## 2. Materials and Methods

### 2.1. Human Anterior Lens Capsules

Anterior lens capsules with LECs were collected from diabetes patients with cataracts (diabetic cataract (DC) group, *n* = 18 eyes) and patients with age-related cataracts (control (CON) group, *n* = 18 eyes) during cataract surgery. The characteristics of all the patients are listed in Table 1. All donors provided written informed consent, and the protocol was performed in accordance with the principles of the Declaration of Helsinki and approved by the Institutional Research Ethics Committee of the Fourth Military Medical University.

### 2.2. Antibodies

Primary and secondary antibodies used in this study are listed in Table 2.

### 2.3. Cell Culture

In human LECs, HLE-B3 (human lens epithelial, ATCC CRL-11421TM) cells were purchased from American Type Culture Collection (ATCC, Manassas, VA, USA). The cell line was authenticated by Qingke Biological Technology Company via short tandem repeat (STR) profiling to verify the human unique DNA profile and rule out intraspecies contamination. The STR analysis was performed on 20 core STR loci.

HLE-B3 cells were cultured in Dulbecco's modified Eagle medium (DMEM) supplemented with 10% fetal bovine serum (HyClone, Logan, Utah, USA), 100 mg/ml streptomycin, and 100 IU/ml penicillin (Gibco, Grand Island, New York, USA), and were incubated at 37°C in a humidified atmosphere containing 5% CO_2_. Culture medium was replaced every two days. When the cells were at 75–80% confluence, they were treated with 0.25% trypsin-0.02% EDTA solution and passaged. Cell number was counted by an automated cell counter (Ruiyu, Shanghai, China).

HLE-B3 cells were divided randomly into a normal control group (NC, 5.5 mM glucose) and high-glucose groups (25.5 mM glucose and 35.5 mM glucose). In the NC group, HLE-B3 cells were cultured in DMEM with 5.5 mM glucose, while in the high-glucose groups, the cells cultured in DMEM were treated with 20 mM glucose and 30 mM glucose, respectively, for 24 h and 48 h.

### 2.4. Cell Transfection

miR-144-3p mimics negative control (NC), miR-144-3p mimics, miR-144-3p inhibitor negative control (NC), miR-144-3p inhibitor, pcDNA3.1, and pcDNA3.1-*MALAT*1 were synthesized by RiboBio (Guangzhou, China). Sh*MALAT*1 negative control (NC), sh*MALAT*1, sh*NRF*2, and sh*NRF*2 negative control (NC) were purchased from GenPharma (Shanghai, China). When the confluence of HLE-B3 cells that were cultured in DMEM with 5.5 mM glucose reached 70%–80%, the cells were transfected with above RNA fragment by Lipofectamine 3000 (Invitrogen, Carlsbad, CA, USA) for 36 h and then were stimulated with 30 mM glucose for 24 h.

### 2.5. RNA Isolation and qRT-PCR

Total RNA was extracted by TRIzol reagent (Invitrogen, Carlsbad, CA, USA) according to the manufacturer's protocol. RNAs in the cytoplasm and nucleus were extracted by the Cytoplasmic & Nuclear RNA Purification Kit (Norgen, Belmont, CA, USA). The RNA was reverse-converted into cDNA in accordance with instructions using the reverse transcription kit (RiboBio, Guangzhou, China). qRT-PCR was performed with SYBR Green master mix (Takara, Japan), and then the relative level of RNA was detected by the Applied Biosystems StepOnePlus Real-Time PCR System (Applied Biosystems, Singapore). The primer sequences are listed in Table 3.

*β*-Actin was used as total and cytoplasmic controls, and U6 was used as nuclear control, respectively.

### 2.6. Western Blot Analysis

In brief, HLE-B3 cells were harvested and lysed by cell lysis buffer (Beyotime, Shanghai, China). Proteins in the cytoplasm and nucleus were extracted by cytoplasmic nuclear separation kit (Beyotime, Shanghai, China). Protein concentration was determined by BCA protein detection reagent (Beyotime, Shanghai, China). 30 *μ*g/lane proteins were loaded on 10% or 15% SDS-PAGE gel for protein separation, after which the separated proteins were transferred to PVDF membranes. After being blocked at room temperature for 1 h, the membranes were probed with the primary antibodies (anti-ZO-1, anti-E-cadherin, anti-FN, anti-NRF2, anti-KEAP1, anti-*α*-SMA, anti-*β*-tubulin, anti-NICD, and anti-Snail) at 4°C overnight. After being incubated with HRP-conjugated secondary antibody for 1 h, the protein bands were visualized with enhanced chemiluminescence (ECL, ZETA, Beijing, China). Relative expressions of proteins were determined by densitometer and expressed as absorbance units. Each experiment was repeated three times.

### 2.7. Dual-Luciferase Assay for Promoter Activity

The putative miRNA binding sites of MALAT1 were predicted by LncBase v2.0 (http://carolina.imis.athena-innovation.gr/diana_tools/web/index.php?r=lncbasev2/index-predicted), miRDB (http://mirdb.org/), and TargetScan (http://www.targetscan.org/mamm_31/). miR-144-3p was the most highly conserved miRNA of all putative miRNAs in mammals. The corresponding mutants were created by mutating the binding site of hsa-miR-144-3p seed region.

Cotransfections were completed by Lipofectamine 3000 in HLE-B3 cells, such as pmiR-RB-Report™ h-MALATI1-WT (MALAT1-WT, wild-type), pmiR-RB-Report™ h-MALAT1-MUT (MALAT1-MUT, mutant-type), pmiR-RB-Report™ h-NRF2-WT (NRF2-WT, wild-type), and pmiR-RB-Report™ h-NRF2-MUT (NRF2-MUT, mutant-type) combined with miR-144-3p mimics, miR-144-3p inhibitor, miR-144-3p mimics negative control (NC), and miR-144-3p inhibitor negative control (NC). The culture medium was removed at 48 h after cotransfection, and then 35 *μ*l 1× phosphate-buffered saline (PBS) and 35 *μ*l luciferase substrate were added. After incubation for 10 min, the medium was transferred to a white cell culture plate LUMITRAC™ 200 well, and firefly luciferase fluorescence values were determined by the dual-luciferase reporter assay system (Promega, Fitchburg, Wisconsin, USA). Subsequently, 30 *μ*l stop reagent was added for 10 min, and the Renilla luciferase fluorescence value was detected.

### 2.8. Transwell Assay

Transwell chambers with 8 *μ*m pore inserts (Millipore, Danvers, Massachusetts, USA) were used to investigate cell migration. For cell migration assay, 2 × 10^4^ HLE-B3 cells or different transfected HLE-B3 cells were seeded in the upper chambers with 200 *μ*l of serum-free DMEM (5.5 mM glucose), while in the lower chambers, the medium was 600 *μ*l DEME in addition to 30 mM glucose (35.5 mM glucose) and 10% fetal bovine serum. After 24 h incubation, the cells in the upper chamber were carefully wiped off with a cotton swab. Next, the filters were fixed in 4% paraformaldehyde for 30 min and stained with 0.1% crystal violet for 20 min. Stained cells in five random fields per chamber were counted under an inverted microscope (Olympus, Nagoya, Japan). Every experiment was repeated three times independently.

### 2.9. Wound Healing Assay

Wound healing assays were performed to detect cell migration. First, 1 × 10^5^ HLE-B3 cells, or different transfected HLE-B3 cells, were seeded in six-well plates and reached 100% confluence overnight. A pipette tip of 1 ml was used to scratch the monolayer cells to make an artificial wound. After rinsing with PBS three times to remove the detached cells, the cells on the plates were cultured in DMEM with 5.5 mM glucose or 35.5 mM glucose for 24 h. Wound width was detected before treatment (0 h) and 24 h after treatment. Images were obtained by an inverted microscope (Olympus, Nagoya, Japan). Woundclosure = woundwidth(0h) − woundwidth(24h)/woundwidth(0h).

### 2.10. Immunofluorescence Staining Assay

Human anterior lens capsules with lens epithelial cells obtained from the cataract surgery were fixed with 4% polyformaldehyde immediately for 30 min. HLE-B3 cells were cultured and treated in a 12-well plate with a cover glass to prepare the cell slide. After different treatments, the cells on the cover glass were fixed with 4% polyformaldehyde for 30 min. The capsular specimens and cultured cells were washed with PBS three times, 5 min for each wash, and then treated with 1% Triton X-100 in PBS for 30 min to enhance the permeability of the cell membranes. In order to reduce the nonspecific immunoreactions, 1% bovine serum albumin in PBS was dropped on the cells for 30 min at room temperature, and then the capsules and cultured cells were incubated with the primary antibodies (anti-*α*-SMA, anti-E-cadherin, and anti-NRF2 for capsules; anti-FN, anti-NRF2, and anti-Snail for cultured cells) at 4°C overnight. After incubation with fluorophore-conjugated secondary antibody for 1 h, the cell nuclei were stained with 4′,6-diamidino-2-phenylindole (DAPI).

Under the confocal scanning laser microscope (FV1000, Nagoya, Olympus, Japan), the stained cells were observed and the images were acquired. The information about primary and secondary antibodies used in this article is listed in Table 2.

### 2.11. ROS Detection Assay

Transfected HLE-B3 cells were incubated with 2′,7′-dichlorofluorescein diacetate (DCFH-DA) (MCE, New Jersey, USA) for 30 min. After washing with cold PBS three times, the cells were observed, and the images were acquired under the confocal scanning laser microscope (FV1000, Nagoya, Olympus, Japan). The cells stained by DCFH-DA were harvested and washed with cold PBS, and then suspended in 1 ml PBS. The fluorescence intensity of the cells was measured by flow cytometry (BD, San Jose, CA, USA).

### 2.12. Cell Cycle Assay

The treated cells were washed by cold PBS and fixed with 70% alcohol for 4 h. Then, the cells were stained with propidium iodide for 10 min at 37°C in darkness. Flow cytometry (BD, San Jose, CA, USA) was used to detect the percentage of cells in G0/G1, S, and G2/M phases.

### 2.13. Statistical Analysis

All data were expressed as the mean ± standarddeviation and analyzed with SPSS statistical analysis software, version 18.0 (Chicago, IL, USA). For comparisons of multiple groups at different time points, statistical analysis was performed using repeated measures analysis of variance followed by Tukey's post hoc test. For groups at the same time point, Student's *t*-test was used. *P* < 0.05 was considered to indicate a statistically significant difference.

## 3. Results

### 3.1. The Expression of *FN*, *α-SMA*, *E-cadherin*, and *ZO-*1 in the Lens Epithelium of Diabetic Cataracts and High-Glucose-Treated HLE-B3 Cells

**Figure 1:**
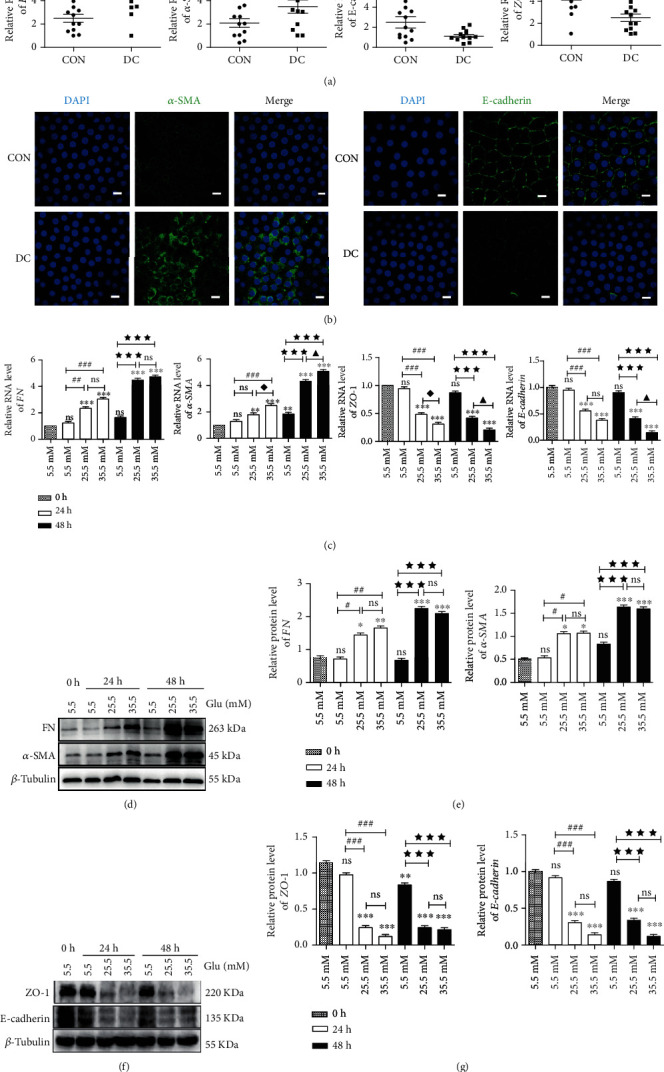
Expressions of EMT markers in lens epithelial cells of diabetic patients and HLE-B3 cells stimulated by high glucose. (a) The expression of *FN*, *α-SMA*, *E-cadherin*, and *ZO*-1 in lens epithelial cells of patients with age-related cataracts (control group, *n* = 12) and diabetic patients with cataracts (diabetic cataract group, *n* = 12). Anterior lens capsules were obtained and analyzed by qRT-PCR, using *actin* as the internal control. HLE-B3 cells were cultured in complete DMEM containing 5.5, 25.5, and 35.5 mM glucose for 24 h or 48 h. (b) Immunofluorescent images of *α*-SMA and E-cadherin in lens epithelial cells on anterior lens capsules. Green represents *α*-SMA and E-cadherin staining, respectively, and blue represents nuclear DNA staining by DAPI, bars = 10*μ*m. (c) RNA expression of *FN*, *α-SMA*, *E-cadherin*, and *ZO-*1 in HLE-B3 cells was determined by qRT-PCR, using *actin* as the internal control. (d) The expressions of FN and *α*-SMA in HLE-B3 cells, using *β*-tubulin as the internal control, and quantified (e). *n* = 3. (f) The expressions of ZO-1 and E-cadherin in HLE-B3 cells, using *β*-tubulin as the internal control, and quantified (g). *n* = 3. ns means *P* > 0.05, one symbol means *P* < 0.05, two symbols mean *P* < 0.01, and three symbols mean *P* < 0.001. *n* = 3. ☆ *vs.* diabetic cataract group, ∗*vs.* group 5.5 mM at 0 h, # *vs.* group 5.5 mM at 24 h, ◆ *vs.* group 25.5 mM at 24 h, ★ *vs.* group 5.5 mM at 48 h, and ▲ *vs.* group 25.5 mM at 48 h.

Compared with age-related cataracts (CON), the expressions of *α*-SMA and FN on RNA level in the lens epithelium of diabetic cataracts (DC) were significantly increased; meanwhile, the expressions of epithelial cell markers *E-cadherin* and *ZO-*1 were significantly decreased (Figure 1(a)). Immunofluorescence staining assay showed that the expression of *α*-SMA was higher in diabetic cataract lens epithelium tissues. However, the epithelial cell marker E-cadherin was lower in lens epithelium of diabetic cataracts compared to age-related cataracts (Figure 1(b)).

In HLE-B3 cells, qRT-PCR and western blot analysis showed that the expressions of *α*-SMA and FN increased gradually and significantly with the concentration (25.5 mM and 35.5 mM) and duration of high glucose (24 h and 48 h) on both RNA and protein levels compared with the control group (5.5 mM); meanwhile, the expressions of epithelial cell markers E-cadherin and ZO-1 were significantly decreased (Figures 1(c)–1(g)). These results suggested the occurrence of EMT in human LECs of diabetic cataracts.

### 3.2. Increased Expression Levels of *MALAT*1 and *NRF*2 in the Lens Epithelium of Diabetic Cataracts and High-Glucose-Treated HLE-B3 Cells

**Figure 2:**
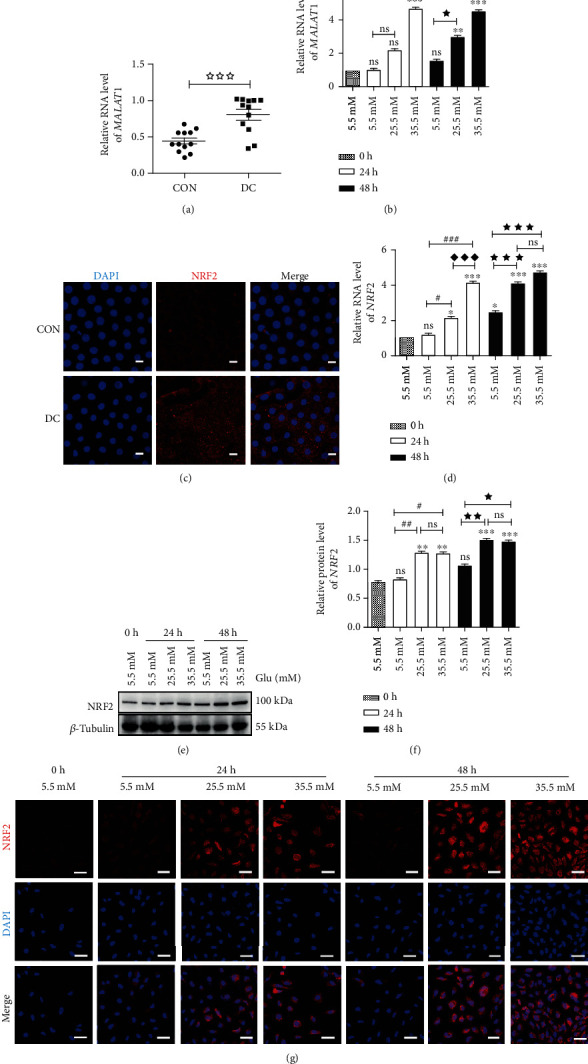
Elevated expressions of *MALAT*1 and NRF2 in lens epithelial cells of diabetic patients and HLE-B3 cells stimulated by high glucose. (a) *MALAT*1 expression in lens epithelium of diabetic cataracts and (b) HLE-B3 cells induced by high glucose, using *actin* as the internal control. (c) Immunofluorescent images of NRF2 in lens epithelial cells on anterior lens capsules. Red represents NRF2 and blue represents nuclear DNA staining by DAPI, bars = 10*μ*m. (d) *NRF*2 expression in HLE-B3 cells induced by high glucose, using *actin* as the internal control. (e) The expressions of NRF2 in HLE-B3 cells, using *β*-tubulin as the internal control, and quantified (f). *n* = 3. (g) Immunofluorescent images of NRF2. Red represents NRF2 staining and blue represents nuclear DNA staining by DAPI, bars = 50*μ*m. ns means *P* > 0.05, one symbol means *P* < 0.05, two symbols mean *P* < 0.01, and three symbols mean *P* < 0.001. *n* = 3. ☆ *vs.* diabetic cataract group, ∗*vs.* group 5.5 mM at 0 h, # *vs.* group 5.5 mM at 24 h, ◆ *vs.* group 25.5 mM at 24 h, ★ *vs.* group 5.5 mM at 48 h, and ▲ *vs.* group 25.5 mM at 48 h.

The expression of *MALAT*1 level in the LECs of diabetic cataracts was significantly increased compared with age-related cataracts (CON) (Figure 2(a)). In HLE-B3 cells stimulated by high glucose, the expression of *MALAT*1 increased significantly with the concentration of glucose (25.5 mM and 35.5 mM) compared with the control group (5.5 mM) at 24 h and was persistent at 48 h (Figure 2(b)).

Immunofluorescence staining assay showed that the expression of NRF2 was higher in lens epithelium of diabetic cataracts compared with age-related cataracts (Figure 2(c)). In HLE-B3 cells, the expression of NRF2 also elevated gradually with increasing glucose concentration on both protein and RNA levels compared with the control group (Figures 2(d)–2(f)). This was confirmed by the data showing the increase of NRF2 fluorescence intensity in the immunofluorescence staining assay (Figure 2(g)). Based on the above data, we selected the treatment of 35.5 mM glucose and 24 h for the following experiments.

### 3.3. Downregulation of *MALAT*1 in HLE-B3 Cells in a Hyperglycemic Environment Suppressed ROS Production, NRF2 Expression, and Its Nucleus Translocation, Arrested S Phase Cell Cycle, and Attenuated EMT

**Figure 3:**
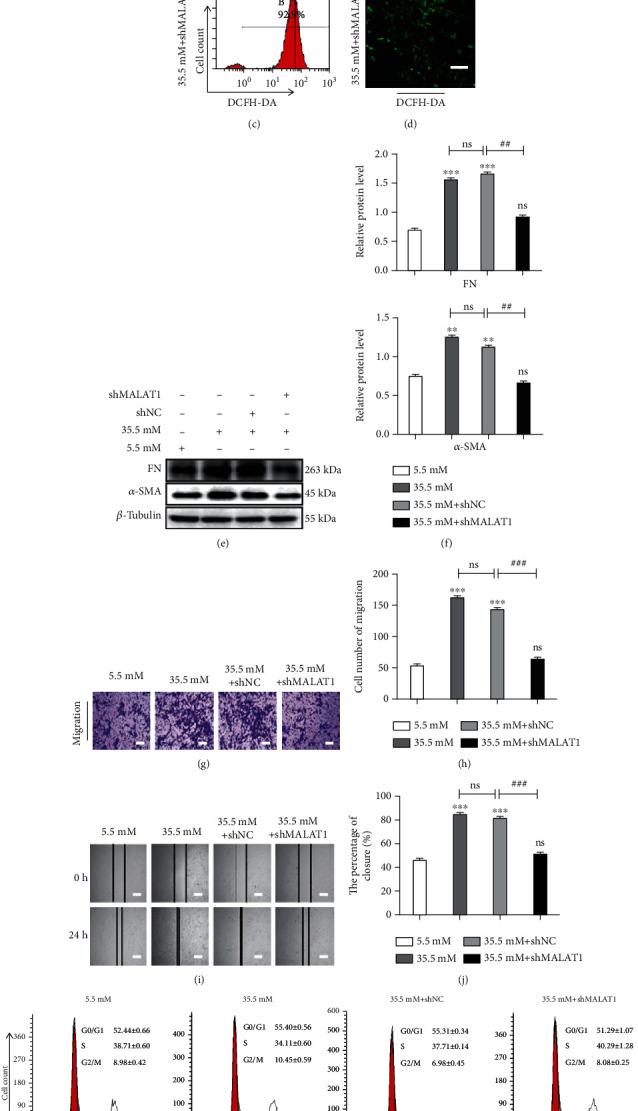
*MALAT*1 regulates production of ROS, expressions of FN, *α*-SMA, and NRF2, and nuclear translocation of NRF2 in HLE-B3 cells stimulated by high glucose. After transfection with sh*MALAT*1 for 36 h, HLE-B3 cells were cultured in DMEM containing 5.5 and 35.5 mM glucose for 24 h. (a) After transfection with four different sh*MALAT*1s for 36 h, *MALAT*1 expression is determined by qRT-PCR in HLE-B3 cells. (b) ROS generation in HLE-B3 cells induced by high glucose detected by flow cytometric analysis (c) and immunofluorescence staining with DCFH-DA (d) (bars = 100*μ*m). (e) The expressions of FN and *α*-SMA in HLE-B3 cells, using *β*-tubulin as the internal control, and quantified (f). *n* = 3. After transfection with sh*MALAT*1, the migration ability of HLE-B3 cells stimulated by high glucose was detected by Transwell assay (g, h), bars = 100*μ*m, and wound healing assay (i, j), bars = 500*μ*m. (k) Cell cycle transition in HLE-B3 cells and quantification (l). (m) The protein expression of NRF2 and nuclear translocation were detected by western blot with *β*-tubulin and histone as the internal control, and quantified (n). *n* = 3. ns means *P* > 0.05, one symbol means *P* < 0.05, two symbols mean *P* < 0.01, and three symbols mean *P* < 0.001. *n* = 3. ∗*vs.* 5.5 mM group at 24 h and # *vs.* 35.5 mM+shNC group.

It has been reported that ROS plays an important role in the pathogenesis of EMT [34, 35]. To define whether upregulated *MALAT*1 is associated with production of ROS in high-glucose-treated HLE-B3 cells, we knocked down *MALAT*1 by transfection with sh-*MALAT*1 in the cells. As shown in Figure 3(a), among the four sh-*MALAT*1s prepared, the first sh*MALAT*1 was the most effective at downregulating *MALAT*1, as revealed by qRT-PCR. So therefore, the first sh*MALAT*1 was selected for the following experiments. After treatment with high glucose for 24 h, ROS levels in the cells increased significantly as shown by flow cytometry and immunofluorescence assays, and notably could be inhibited by sh*MALAT*1 (Figures 3(b)–3(d)), suggesting that knockdown of *MALAT*1 was capable of preventing the production of ROS in HLE-B3 cells.

When sh*MALAT*1 was transfected in high-glucose-induced HLE-B3 cells, the elevated expressions of FN and *α*-SMA were suppressed significantly (Figures 3(e) and 3(f)). In addition, the cell migration of high-glucose-stimulated cells increased significantly with Transwell and wound healing assays, while the transfection of sh*MALAT*1 abolished the migration of HLE-B3 cells in high glucose conditions (Figures 3(g)–3(j)).

Cell cycle analysis demonstrated that the cells could be retarded in G0/G1 phase after treatment with high glucose. While after transfection with *MALAT*1, the percentage of cell in S phase increased and the percentage in G0/G1 phase reduced compared to NC (Figures 3(k) and 3(l)).

In this set of experiments, western blot assay showed that the expressions of both total NRF2 and NRF2 in the nucleus increased in high-glucose-treated HLE-B3 cells, which could be prevented by the transfection of sh*MALAT*1 (Figures 3(m) and 3(n)).

Collectively, the above data suggested that downregulation of *MALAT*1 in HLE-B3 cells under high glucose conditions suppressed the generation of ROS, the expression, and nucleus translocation of NRF2, released cell cycle inhibition, and attenuated EMT and cell migration.

### 3.4. Downregulation of *NRF*2 Prevented EMT and Cell Migration in High-Glucose-Treated HLE-B3 Cells

**Figure 4:**
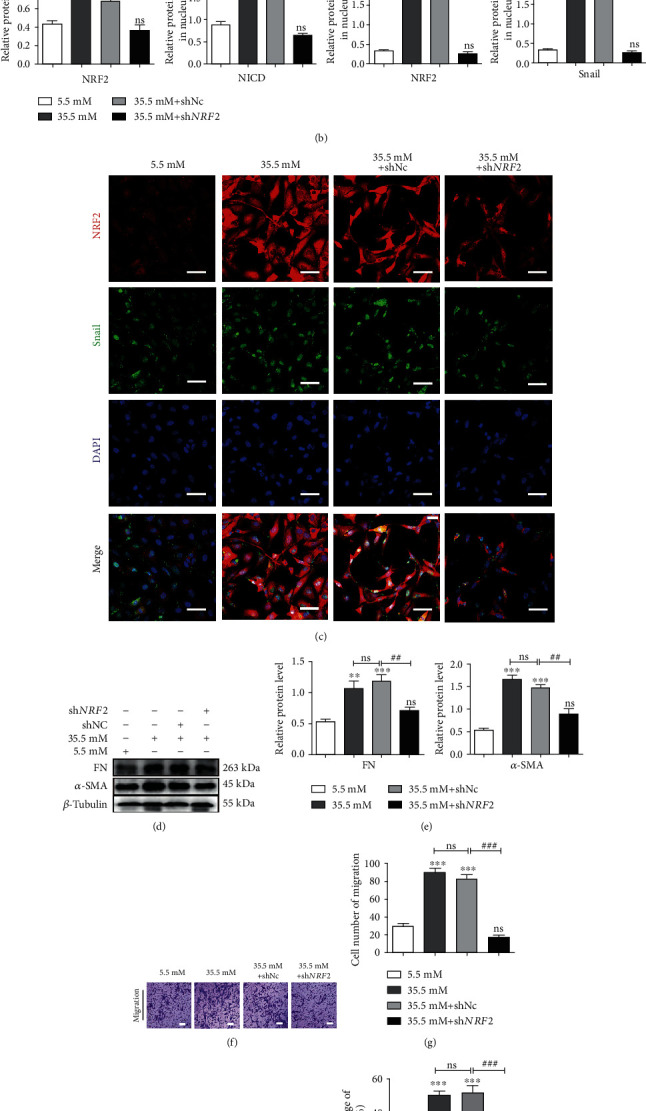
Downregulation of NRF2 inhibits EMT and migration of HLE-B3 cells stimulated by high glucose through the NRF2/Notch1/Snail pathway. After transfection with sh-NRF2 for 36 h, HLE-B3 cells were cultured in complete DMEM containing 5.5 and 35.5 mM glucose for 24 h. (a) The expressions of NRF2, NICD, and Snail were detected by western blot with *β*-tubulin and histone as the internal control, and quantified (b). *n* = 3. (c) The expressions of NRF2 and Snail by immunofluorescent staining. Green represents Snail, red represents NRF2, and blue represents nuclear DNA staining by DAPI, bars = 50*μ*m. (d) The expressions of FN and *α*-SMA in HLE-B3 cells, using *β*-tubulin as the internal control, and quantified (e). *n* = 3. After transfection of sh*MALAT*1, the migration ability of HLE-B3 cells was detected by Transwell assay (f, g), bars = 100*μ*m, and wound healing assay (h, i), bars = 500*μ*m. ns means *P* > 0.05, one symbol means *P* < 0.05, two symbols mean *P* < 0.01, and three symbols mean *P* < 0.001. *n* = 3. ∗*vs.* 5.5 mM group and # *vs.* 35.5 mM+shNC group.

Previous studies have shown that NRF2 contributed to EMT through activation of the Notch signaling pathway [25]. Notch1 is one of the transmembrane Notch family receptors, which can drive Notch signaling when combined with the Rbpj*κ* transcription factor. After canonically accepting ligands, the receptor undergoes cleavage to yield the NICD that translocates to the nucleus. It has been clearly shown that NRF2 can activate the Notch1 pathway [36]. The Notch1 signaling pathway can regulate the process of EMT by regulating the Notch1/Snail signaling pathway [37, 38]. The efficacy of sh*NRF*2 on knockdown of NRF2 in high-glucose-treated HLE-B3 cells was confirmed with western blot and immunofluorescence staining assays (Figures 4(a)–4(c)). We found that the transfection of sh*NRF*2 could reduce the expression levels of NICD and Snail in the nucleus of HLE-B3 cells under high glucose conditions (Figures 4(a) and 4(b)). The reduction of Snail in the nucleus was confirmed by immunofluorescence staining assay (Figure 3(c)). Importantly, knockdown of *NRF*2 could suppress the expressions of *α*-SMA and FN (Figures 4(d) and 4(e)) and prevent cell migration in high-glucose-treated HLE-B3 cells (Figures 4(f)–4(i)).

Taken together, NRF2 may function as one of the downstream of *MALAT*1 in regulating EMT in high-glucose-treated HLE-B3 cells via the Notch/Snail pathway.

### 3.5. *MALAT*1 Regulated *NRF*2 through miR-144-3p in EMT of High-Glucose-Stimulated HLE-B3 Cells

**Figure 5:**
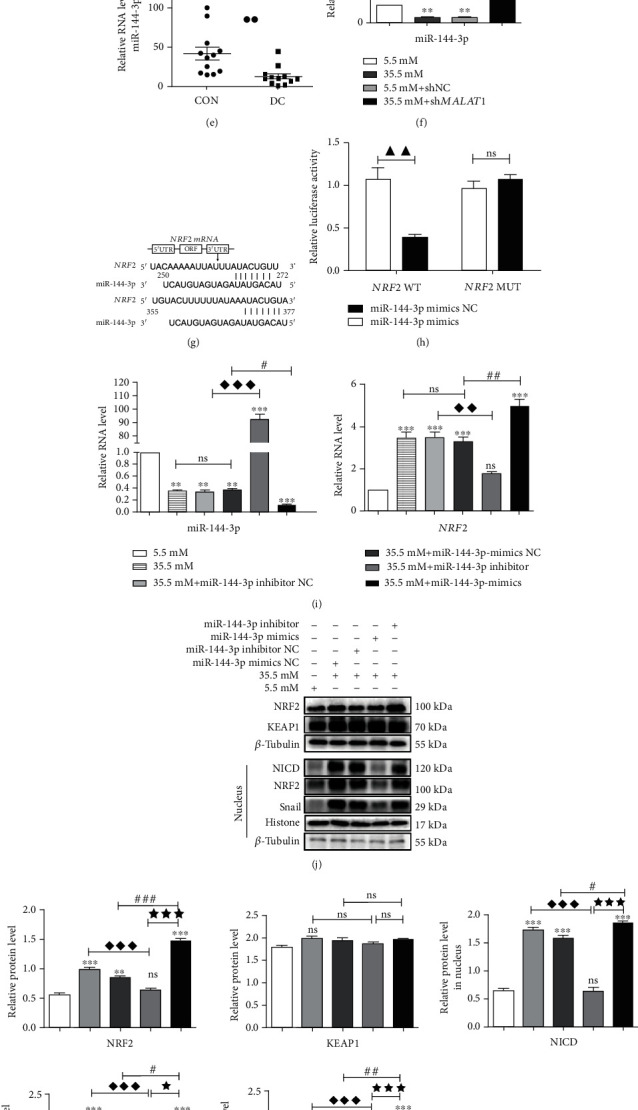
*MALAT*1 regulates NRF2 expression via sponge miR-144-3p in HLE-B3 cells. (a) qRT-PCR analysis of *MALAT*1 expression in the cytoplasm and nucleus of HLE-B3 cells. (b, c) Prediction of targets between *MALAT*1, *NRF*2, and miR-144-3p by TargetScan and LncBase v.2.0 software. (d) The interaction between *MALAT*1 and miR-144-3P is verified by dual-luciferase reporter gene assays. (e) Quantification expression of miR-144-3p in lens epithelial cells on anterior capsules using *U*6 as the internal control. (f) After transfection of sh*MALAT*1, quantification expression of miR-144-3p in HLE-B3 cells used *U*6 as the internal control. (g) Prediction of target relationship between *NRF*2 and miR-144-3p by miRBase and TargetScan. (h) The interaction between *NRF*2 and miR-144-3p was verified by dual-luciferase reporter gene assays. (i) After separately transfection of miR-144-3p mimics, miR-144-3p inhibitor, miR-144-3p mimics NC, or miR-144-3p inhibitor NC, quantification of RNA expression of miR-144-3p and *NRF*2 in HLE-B3 cells. (j) The expressions of NRF2, NICD, KEAP1, and Snail were detected by western blot with *β*-tubulin and histone as the internal control, and quantified (k). *n* = 3. (l) The expressions of FN and *α*-SMA in HLE-B3 cells, using *β*-tubulin as the internal control, and quantified (m). *n* = 3. ns means *P* > 0.05, one symbol means *P* < 0.05, two symbols mean *P* < 0.01, and three symbol mean *P* < 0.001. *n* = 3. ∗*vs.* 5.5 mM group, ◆ *vs.* 35.5 mM+miR-144-3p mimics NC group, *# vs.* 35.5 mM+miR-144-3p inhibitor NC group, and ★ *vs.* miR-144-3p mimics group.

Here, by performing nuclear-cytoplasmic fractionation assay, we found that MALAT1 was predominately located in the cytoplasm in HLE-B3 cells (Figure 5(a)).

Previous studies have demonstrated that lncRNAs can sponge miRNAs, thus reducing the miRNA-induced repression of their target mRNAs [28, 39]. To predict which miRNA may be included in the “sponge” and regulate *NRF*2 expression, we used the online bioinformatics prediction software miRDB, TargetScan, and LncBase v.2.0. We found five broadly conserved miRNAs (hsa-miR-27-3p, hsa-miR-140-5p, hsa-miR-28-5p, hsa-miR-708-5p, and hsa-miR-144-3p) that might interact with both *MALAT*1 and *NRF*2 (Figures 5(b) and 5(c)). Because miR-144-3p is downregulated in fat production and in the retina and liver of diabetes [28, 31, 33, 40], we selected it in the following experiments.

Dual-luciferase reporter assay showed that cotransfection of miR-144-3p mimics and wild-type (WT) *MALAT*1 reduced the fluorescence activity, but cotransfection with mutant (MUT) *MALAT*1 did not (Figure 5(d)), suggesting that miR-144-3p is negatively regulated by *MALAT*1 expression. Compared with the age-related cataracts (CON) and HEL-B3 in the normal-glucose group, the expression levels of miR-144-3p were lower than those in the diabetic cataract (DC) group or in the cells of the high-glucose group (Figures 5(e) and 5(f)).

Dual-luciferase reporter assay also showed that miR-144-3p mimics were able to markedly suppress luciferase expression in the NRF2 WT group, but did not suppress the MUT group (Figures 5(g) and 5(h)). The mRNA level of *NRF*2 was downregulated in the miR-144-3p mimics group compared with the miR-144-3p mimics NC group (Figure 5(i)), suggesting that miR-144-3p negatively regulated *NRF*2 expression.

To investigate the role of miR-144-3p on NRF2 expression and EMT in high-glucose-treated HLE-B3 cells, we examined the expressions of FN, NRF2, *α*-SMA, and KEAP1 in the cells and furthermore the expressions of NRF2, NICD, and Snail in the nucleus. The expression of total NRF2 and expressions of NRF2, NICD, and Snail in the nucleus were all downregulated in high-glucose-stimulated HLE-B3 cells treated with the miR-144-3p mimics. In contrast, these protein expressions could be upregulated by miR-144-3p inhibitor. Interestingly, the expression of KEAP1 had no obvious changes between these groups (Figures 5(j) and 5(k)). In addition, the expressions of FN and *α*-SMA were downregulated by miR-144-3p mimics and were upregulated by miR-144-3p inhibitor in high-glucose-stimulated HLE-B3 cells (Figures 4(l) and 4(m)).

Thus, it is concluded that *MALAT*1 could negatively regulate miR-144-3p, and through the negative regulation of miR-144-3p on NRF2, it could be involved in EMT of HEL-B3 cells in a high-glucose environment.

### 3.6. *MALAT*1 Promoted EMT and Cell Migration through Reducing miR-144-3p and Activating the NRF2/Notch1/Snail Signaling Pathway

**Figure 6:**
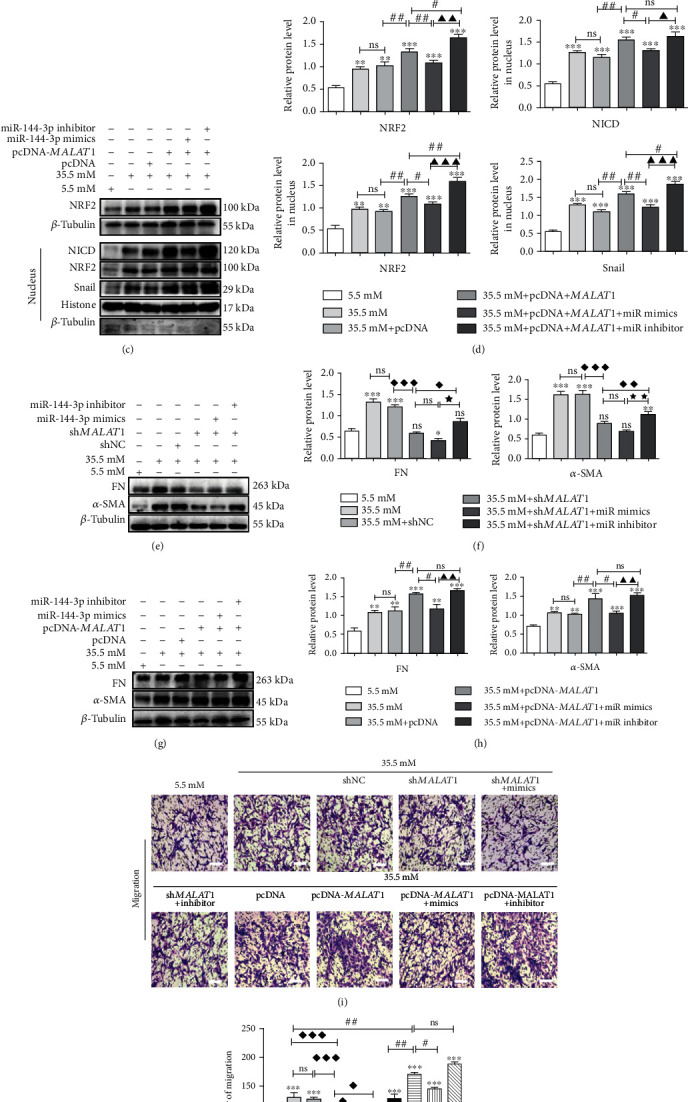
*MALAT*1 promotes EMT and migration via sponge miR-144-3p through the NRF2/Notch1/Snail pathway in HLE-B3 cells. After cotransfection of miR-144-3p mimics, miR-144-3p inhibitor, miR-144-3p mimics NC, or miR-144-3p inhibitor NC with shNC, sh*MALAT*1, pcDNA3.1, and pcDNA3.1-*MALAT*1 quantification of miR-144-3p and NRF2 RNA expression in HLE-B3 cells, HLE-B3 cells were cultured in complete DMEM containing 5.5 and 35.5 mM glucose for 24 h. (a, c) The expressions of NRF2, NICD, and Snail were detected by western blot with *β*-tubulin and histone as the internal control, and quantified (b, d). *n* = 3. (e, g) The expressions of FN and *α*-SMA were detected by western blot with *β*-tubulin as the internal control, and quantified (f, h). *n* = 3. After cotransfection, the migration ability of HLE-B3 cells stimulated by high glucose was detected by Transwell assay (i, j), bars = 100*μ*m. ns means *P* > 0.05, one symbol means *P* < 0.05, two symbols mean *P* < 0.01, and three symbols mean *P* < 0.001. *n* = 3. ∗*vs.* 5.5 mM group, ◆ *vs.* 35.5 mM+sh*MALAT*1 group, ★ *vs.* 35.5 mM+sh*MALAT*1+miR-144-3p mimics group, # *vs.* 35.5 mM+pcDNA3.1-*MALAT*1 group, and ▲ *vs.* 35.5 mM+pcDNA3.1-*MALAT*1+miR-144-3p mimics group.

First, we found that transfection of sh*MALAT*1 in high-glucose-treated HLE-B3 cells resulted in downregulation of total NRF2 in cells as well as downregulation of NRF2, NICD, and Snail in the nucleus compared with levels in the transfection with the sh*MALAT*1 *NC* group and high-glucose group. Conversely, suppressions of these proteins could be partially rescued by cotransfecting miR-144-3p inhibitor, but not by miR-144-3p mimics (Figures 6(a) and 6(b)). Furthermore, upregulation of *MALAT*1 by transfecting pcDNA3.1-*MALAT*1 in high-glucose-treated HLE-B3 cells resulted in increases of total NRF2 in cells, as well as NRF2, NICD, and Snail increases in the nucleus compared with those in the transfection with the pcDNA3.1 and high-glucose groups. Conversely, increases of these proteins could be partially reversed by cotransfecting miR-144-3p mimics, but not by miR-144-3p inhibitor (Figures 6(c) and 6(d)).

Additionally, downregulation of both FN and *α*-SMA by transfection of shMALAT1 in high-glucose-treated HLE-B3 cells could be partially rescued by cotransfecting miR-144-3p inhibitor, but not by transfecting miR-144-3p mimics (Figures 6(e) and 6(f)). Upregulation of FN and *α*-SMA by transfection of pcDNA3.1-*MALAT*1 could be partially rescued by cotransfecting miR-144-3p mimics, but not by cotransfecting miR-144-3p inhibitor (Figures 6(g) and 6(h)).

We used Transwell assay to determine whether *MALAT*1 and miR-144-3p could regulate cell migration in high-glucose-treated HLE-B3 cells. Compared with the control groups (transfection of the pcDNA3.1 group and high-glucose group), cell migration was promoted after transfection with pcDNA3.1-*MALAT*1 in high-glucose-treated HLE-B3 cells, which could be significantly reversed by transfection of a miR-144-3p mimics, but not by transfection of a miR-144-3p inhibitor. Compared with cells transfected with sh*MALAT*1 NC, cell migration was inhibited in the cells transfected with sh*MALAT*1, which could be promoted by cotransfecting miR-144-3p inhibitor, but not by cotransfecting miR-144-3p mimics (Figures 6(i) and 6(j)).

In summary, the above data confirmed that *MALAT*1 promoted EMT and cell migration via reducing miR-144-3p and subsequently activating the NRF2/Notch1/Snail signaling pathway.

## 4. Discussion

In the current study, we found that *MALAT*1 promoted the EMT of LECs by negatively regulating miR-144-3p and subsequently activating the ROS/NRF2/Notch1/Snail signaling pathway under high glucose conditions.

*MALAT*1 is widely upregulated in the aqueous humor, lens epithelium, and fibrovascular membranes of diabetic patients [41]. It can promote EMT by activating Wnt, Notch1, and Akt/mTOR signaling in cancer tissues and cells [42–44]. It also can facilitate EMT in HK-2 cells in diabetic nephropathy[45]. Consistent with previous studies, we also found that *MALAT*1 was aberrantly expressed in LECs of diabetic cataract patients and in high glucose conditions.

In this study, we demonstrated that *MALAT*1 could induce ROS production and NRF2 expression in LECs. However, previous studies have shown that *MALAT*1 can reduce the expression of NRF2 and promote the production of ROS [35]. On the contrary, some studies have shown that *MALAT*1 can reduce the production of ROS by activating NRF2 [46]. The role of *MALAT*1 in regulating NRF2 and ROS seems controversial. We think that *MALAT*1 may control the ROS level in balance by promoting NRF2 expression. ROS are highly expressed in LECs during the onset of diabetic cataracts stimulated by high glucose [18, 21], and could induce EMT by activating several transcription factors, such as Snail, STAT3, and ZEB1, which can be activated by the Notch, G-CSF, MAPK, and PI3K/Akt signaling pathways in cancer cells [47]. It is well known that the classic signaling pathway of KEAP1/NRF2 is the main approach against ROS [48]. KEAP1 binds with NRF2 by acting as an adaptor protein and is a negative regulator of NRF2. Under normal ROS conditions, this interaction is a short incident, with NRF2 exhibiting a short half-life of 13–21 min [49, 50]. While under hyperactive ROS conditions, because of modification of critical cysteine residues in KEAP1 or NRF2 coupled with phosphorylation, NRF2 can detach from KEAP1 and then translocate into the nucleus to promote the transcription of target genes by binding to promoter regions [21].

NRF2 can also be regulated by posttranslational, transcriptional, translational, and epigenetic mechanisms, as well as by several microRNAs [51]. In recent years, many studies have shown that ROS can induce NRF2 into the nucleus to decrease the sensitivity of cancer to radiotherapy and increase the metastasis of cancer [25, 52–54]. NRF2 expression is low in both epithelial and mesenchymal cells, while NRF2 expression is high in mixed epithelial and mesenchymal phenotypes, suggesting that the highly expressed NRF2 in cells may activate EMT [52]. Excessive ROS can lead to apoptosis in LECs, while NRF2 may reduce apoptosis through antioxidant production [19, 55]. In our study, we found that high-glucose-induced ROS may promote NRF2 nucleus translocation. Increased nucleus translocation of NRF2 can promote EMT in LECs, which may occur through the Notch1/Snail pathway. So this study indicates that the increase of NRF2 expression may not always be beneficial in the prevention of cataracts.

Previous studies have indicated that *MALAT*1 can induce EMT in cancer cells by acting as ceRNAs to sink miR-141, miR-3064-5p, miR-200b, and miR-144-3p [28, 29, 56, 57]. NRF2 can be negatively regulated by miRNAs, including miR-153, miR-27a, miR-142-5p, and miR-144-3p, through direct repression of NRF2 messenger RNA in a KEAP1-independent manner, or by enhancing KEAP1 expression. In the present study, we used bioinformatics software to predict that *MALAT*1 and *NRF*2 shared the same miR-144-3p binding sites. Additionally, *MALAT*1 can inhibit the expression of miR-144-3p, which can downregulate NRF2 expression independent of KEAP1. The miR-144-3p expression and function in diabetic cataracts have not been reported before. We detected that miR-144-3p was downregulated in LEC lining with anterior lens capsules and in LECs under high glucose conditions. Even though the highly expressed miR-144-3p can inhibit the EMT of tumor tissues and cells by targeting EZH2, PBX3, and MAP3K8 [58–60], we found that EMT could be negatively regulated by miR-144-3p through the ROS/NRF2/Notch1/Snail pathway in high-glucose-stimulated LECs.

## 5. Conclusions

**Figure 7:**
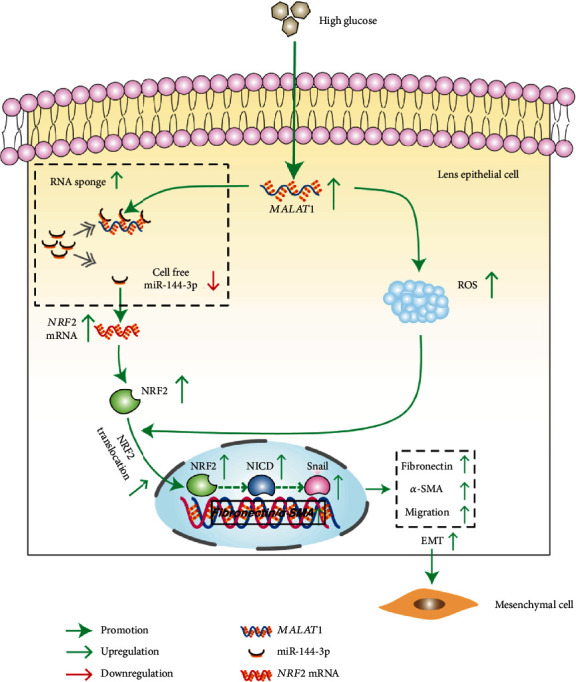
The schematic for the mechanism underlying EMT in high-glucose-treated HLE-B3 cells facilitated by *MALAT*1.

In summary, we have found that *MALAT*1 is upregulated in LECs under high glucose conditions. Upregulated *MALAT*1 can promote ROS production and competitively combine with miR-144-3p. Downregulation of miR-144-3p can promote NRF2 expression. NRF2 could be stimulated by ROS, translocate to the nucleus, and activate the Notch1/Snail pathway, resulting in the EMT of LECs (Figure 7). Therefore, *MALAT*1 may be a potential target for the prevention and treatment of diabetic cataracts.

## Figures and Tables

**Table 1:** Characteristics of the diabetes patients with cataracts and patients with age-related cataracts.

	CON (*n* = 18)	DC (*n* = 18)	*P*
Gender (male/female)	8/10	9/9	0.7385
Age	65.94 ± 10.62	64.94 ± 9.72	0.7764
Fast blood glucose	5.12 ± 0.49	8.60 ± 1.365	<0.0001

The gender and age between the CON and DC groups have no statistically significant difference. The blood glucose in the DC group is higher than that in the CON group (*P* < 0.0001).

**Table:** (a) Primary antibodies used in western blot

Antibody name	Host species	Species reactivity	Concentrations	Catalog number	Company
*β*-Tubulin	Rabbit	Mouse, rat, chicken, cow, dog, human	1 : 1000	#2148	CST, America
NICD	Rabbit	Mouse, rat, human	1 : 1000	#4147	CST, America
Histone	Rabbit	Mouse, rat, monkey, human	1 : 1000	#4499	CST, America
Snail	Rabbit	Mouse, rat, monkey, human	1 : 1000	#3879	CST, America
NRF2	Rabbit	Mouse, monkey, human	1 : 1000	#12721	CST, America
KEAP1	Rabbit	Mouse, rat, monkey, human	1 : 1000	10503-2-AP	Proteintech, China
Fibronectin	Rabbit	Mouse, rat, human	1 : 1000	ab45688	Abcam, UK
*α*-SMA	Rabbit	Mouse, rat, chicken, guinea pig, cow, dog, human, pig	1 : 1000	ab5694	Abcam, UK
E-cadherin	Rabbit	Human, mouse	1 : 1000	24E10	CST, America
ZO-1	Rabbit	Human, monkey	1 : 1000	#8193	CST, America

**Table:** (b) Secondary antibody used in western blot

Antibody name	Concentration	Catalog number	Company
Goat anti-rabbit IgG (H+L)	1 : 2000	SA00001-2	Proteintech, China

**Table:** (c) Primary antibodies used in immunofluorescence staining assays

Antibody name	Host species	Species reactivity	Concentrations	Catalog number	Company
*α*-SMA	Rabbit	Mouse, rat, chicken, guinea pig, cow, dog, human, pig	1 : 100	ab5694	Abcam, UK
E-cadherin	Rabbit	Mouse, rat, human	1 : 100	ab231303	Abcam, UK
NRF2	Rabbit	Human	1 : 100	ab62352	Abcam, UK
Snail	Goat	Mouse, rat, human	1 : 100	ab53519	Abcam, UK

**Table:** (d) Secondary antibodies used in immunofluorescence staining assays

Antibody name	Concentrations	Catalog number	Company
Cy3 goat anti-rabbit IgG (H+L), Alexa Fluor 488-conjugated	1 : 100	A10522	Invitrogen, America
Donkey anti-goat IgG (H+L)	1 : 100	A32814	Invitrogen, America
Donkey anti-rabbit IgG (H+L), Alexa Fluor Plus 488	1 : 100	A32790	Invitrogen, America
Donkey anti-rabbit IgG (H+L), Alexa Fluor Plus 647	1 : 100	A32795	Invitrogen, America

**Table 3:** Primers' sequence.

Primers	Sense	Antisense
*MALAT*1	GCCATTTTAGCAACGCAGAA	GACAGCTAAGATAGCAGCAGCACAACT
*NRF*2	TCCAGTCAGAAACCAGTGGAT	GAATGTCTGCGCCAAAAGCTG
*Fibronectin*	GGCTTGAACCAACCTACGGATGAC	TCCTTCTGCCACTGTTCTCCTACG
*α-SMA*	CTGAACCCCAAGGCCAACCG	GACAATCTCACGCTCAGCAGT
*Actin*	TGTTACCAACTGGGACGACA	CTTTTCACGGTTGGCCTTAG
*ZO*-1	CTGGTGAAATCCCGGAAAAATGA	TTGCTGCCAAACTATCTTGTGA
*E-cadherin*	TACAATGCCGCCATCGCTTACAC	TGACGGTGGCTGTGGAGGTG
*miR-*144-3*p*	Synthesized by RiboBio (Guangzhou, China; Lot N0108)	
*U*6	Synthesized by Sangon Biotech (Shanghai, China; Lot B532451-0020)	

## Data Availability

All data related to this paper may also be requested from the corresponding author.
